# Holland Gin as a Medicine

**Published:** 1852-11

**Authors:** 


*Holland Gin as a Medicine.*—In our last number we accompanied the publication of a circular on this subject, from our fellow-citizen Udolpho Wolfe, Esq., with a brief commentary, expressive of our own views. Since then we have been employing this agent, and thus far with favorable results. But we are in the receipt of several communications on the subject from medical men, which serve to show that Mr. Wolfe's Aromatic Schiedam Schnapps is very extensively in use, and, in the hands of physicians, is proving itself, as a stimulating diuretic, to be eminently successful, after other medication with this intent had been tried in vain. In one of the cases thus reported, abdominal dropsy has been cured, and the necessity of tapping averted; and in another, a distressing case of gravel, so called, has been entirely removed by the passage of a calculus of considerable size, which is ascribed to the use of only two bottles of this article.

We know not the object of Mr. Wolfe in designating his preparation by the singular uneuphonious name of "*Schnapps*," nor of his denominating it in his advertisements, the "concentrated Tincture of Juniper," instead of perpetuating its ancient title of Holland Gin. It is true that he admits it to be nothing else than the latter article in its pure state, unadulterated by noxious drugs, and hence he contradistinguishes it from Gin of commerce, nearly all of which, as is well known, is manufactured here and elsewhere from inferior whisky, and refuse drugs. The name he has given it, however, may serve the purpose of designating his article, as prepared exclusively for medical purposes, and thus commend it to physicians, for whose convenience it is on sale only by reputable druggists and apothecaries.

As respects its medicinal and curative effects, we understand him to claim only that it is a pure and reliable article of Holland Gin, and as such worthy of the confidence of physicians, in those diseases for which they are wont to prescribe it, and have hitherto only been restrained, by finding it impracticable to obtain the article in a pure state. Nor should any prejudice against alcoholic medicine deprive the afflicted of the benefit of this article, which from time immemorial has held its place among the remedial agencies of the materia medica, if it be found worthy of confidence by continued experience. At all events, those who persist in the employment and toleration of other alcoholic medicines, as tinctures, bitters, &c., and especially those who prescribe gin under any circumstances, must all unite in giving the preference to a pure article over the manifold adulterations so rife in the market. Mr. Wolfe liberally supplies physicians with a sample bottle for analysis and trial, as set forth in his circular, and stakes the reputation of the remedy upon the innocence, safety and efficiency of his Holland Gin, when used under medical advice; and pledges his own character in business that the article will not disappoint any who use it.

We shall take occasion hereafter to discuss the whole subject of alcoholic medicine, a topic which has recently been attracting much attention in Europe and America.—*New York Medical Gazette.*

